# An Eye Tracker Study on the Understanding of Implicitness in French Elementary School Children

**DOI:** 10.3390/brainsci14121195

**Published:** 2024-11-27

**Authors:** Maria Pia Bucci, Aikaterini Premeti, Béatrice Godart-Wendling

**Affiliations:** 1 ICAR UMR 5191 CNRS, ENS de Lyon, Université Lyon 2, 69007 Lyon, France; kpremeti@gmail.com; 2 Neurodiderot, UMR 1141, Université Paris Cité, Hôpital Robert Debré AP-HP, 75019 Paris, France; 3 ISJPS UMR 8103 CNRS, Université Paris 1 Panthéon Sorbonne, 75005 Paris, France; beatrice.godart-wendling@cnrs.fr

**Keywords:** children, implicitness, eye movements, development

## Abstract

Background: The aim of this study is to use an eye tracker to compare the understanding of three forms of implicitness (i.e., presupposition, conversational implicatures, and irony) in 139 pupils from the first to the fifth year of elementary school. Methods: The child was invited to read short texts composed of a context about some characters and a target sentence conveying one of the three kinds of implicitness. After that, there was a comprehension yes/no question to check whether the child had understood the implicit content of the target sentence. At the same time eye, movements were recorded by a remote system (Pro Fusion by Tobii). The number of correct answers, the duration, and the number of fixations on the texts were measured. Results: We showed that children’s reading time is positively correlated with the accurate comprehension of implicitness, and that children similarly understand the three types of implicitness. Furthermore, the number and the duration of fixations depend both on the age of the children and on their good or poor understanding of the implicit contents. This fact is particularly noticeable for children in the first-grade class, for whom fixations are significantly longer and more frequent when they correctly understand sentences containing implicitness. Conclusion: These results argue in favor of the possibility of teaching the comprehension of some types of implicitness (presupposition, implicature, and irony) from an early age.

## 1. Introduction

Understanding a text requires not only grasping its literal meaning but also being able to identify and interpret its implicit meaning. Implicitness plays an important role in comprehension because it structures the coherence of the mental representation that the reader must develop to obtain an accurate interpretation of what is meant in a text [1]. By implicitness, we mean the covert pieces of information that must be inferred (in the broadest sense) from what is written, without having been explicitly stated [2]. Implicit content can be of different kinds and come from a variety of sources (a word, a group of words, a sentence, or a context). To do justice to this diversity, this study will focus on three types of implicit content:

1. Presuppositions, which are characterized by the fact that they are anchored in the literal meaning by being carried by a lexical trigger. For example, it is because the speaker uses the verb “finish” in (1a) that the addressee makes the inference (1b). As one can finish only what was started before, “finish” lexically presupposes “having started”:
  (1) a. Alexander has still not finished painting the walls.
    b. Alexandre had already started painting the walls.
2. Conversational implicatures, which are non-logical inferences we make out of habit. Unlike presuppositions, they are not triggered by particular linguistic forms, but by taking into account the Gricean principle of cooperation and conversational maxims which regulate conversation in their rational use [3]. Therefore, it is because the addressee knows from his knowledge of the world how a body reacts when someone is car sick and has to drive for two hours on bumpy roads, that he will deduce (2a):
  (2) a. Raphaël suffers from carsickness. He has to drive for two hours on small bumpy roads to go shopping.
    b. He might throw up.
3. Irony is a type of implicitness in which the reader must recognize a form of discordance by the narrator between what is said and what is actually meant [4]. By considering the context, the reader infers that the ironic sentence means the opposite of its literal meaning:
  (3) a. Alice wants to help her mother carry her shopping. She takes a packet of tissues. Her mother says to her:
    b. How strong you are!

The few psycholinguistic studies that have investigated children’s understanding of these three types of implicitness agree that children are able to understand them as early as age five. However, all these studies use different methodologies and protocols and base their results on an examination of the comprehension of a limited number of linguistic facts that are discussed from article to article and in different languages.

Thus, for presupposition, studies have mainly focused on triggers such as “only” (for English [5,6]; “seulement” (for French [7]); “also” and “too” (for English: [5]); “ook” (for Dutch [8]); “auch” (for German [6,9,10,11]); “aussi” (for French [7]); “more” (for English); and “encore” (for French) [7]. Some studies on English also focus on some factive verbs such as “regret, know, forget, be sorry, etc.” [12,13].

As far as implicatures are concerned, the studies are more numerous but essentially focused on the understanding of scalar implicatures involving quantification terms such as “some” compared to “all” (for English: [14,15,16,17,18]; for French: “tous” versus “certains”, [15,19]) or the logical disjunction “or” (for English: [20,21]); for French and Japanese: [22]).

The most numerous and varied studies concern irony, but there is a lack of unity in the issues addressed, since the analyses range from the role of intonation and/or context [23,24] to social norms [25] or the choice of default interpretation [26], as well as the link between irony and jokes [27].

To overcome these limitations, we tested children’s understanding using a wide variety of presuppositions (generated by a definite description, a factive verb, an implicative verb, an aspectual adverb, or a connector), conversational implicatures (based on the calculation of the speaker’s intentions, on the interpreter’s knowledge of the world, etc.), and antithetical ironic sentences (see Appendix A).

To the best of our knowledge, no studies exist exploring eye movements during the reading of sentences containing implicit content. Oculomotor recording is a non-invasive technique that allows us to understand the reading process in real-time, contributing to an objective assessment independent of the verbal responses of readers [28].

The oculomotor pattern during reading by children has been well-known for several decades and it differs from that of adult subjects [29,30,31,32,33,34]. For instance, children starting to read use the non-lexical route and they make several fixations with longer durations. With reading experience, the oculomotor pattern changes and children more frequently use the lexical route: fixations also become less frequent and their duration is shortened. This oculomotor behavior is due to an improvement in reading skills, which, for children reading in their mother tongue, is reached at about 10–12 years; at that age, reading is a simple task and the process becomes similar to that in adults.

It is important to note that as previously suggested [35], when reading a text at school, visual attention is dependent on several factors (such as age, gender, low/high attainers, and also the type of lesson (mathematics or English lessons). Not all these points have not been taken into account in our study, given that eye movements were recorded in a room and not in the classroom during lessons.

A recent study [36] compared eye movements in 35 children (aged 10 years old) and 35 adults while reading ironic and literal sentences embedded in a story. For both children and adults, the comprehension of written irony was more difficult than literal text, and even if children reported longer reading times than adults did, the processing of ironic stories was similar between the two groups even if a large variability was observed in the children’s group. The only group difference was that irony comprehension was achieved in children with shorter reading times in contrast to adults. This is the first study recording the processing and comprehension of written irony and eye movements in children.

Another study [37] recorded eye movements in a small group of adult subjects with and without ASD (autism spectrum disorder) (20 per group) while reading a text containing ironic and non-ironic statements. While comprehension was similar in the two groups, subjects with ASD spent more time than control subjects reading and rereading the text in order to construct a coherent understanding of the text.

Based on the above few cited studies combining eye movements and ironic sentences, the goal of the present study was to further explore this issue by recording eye movements in a large number of children and testing not only irony but also other forms of implicitness (such as presupposition and conversational implicatures).

Our hypothesis is firstly that comprehension of implicitness will be age-dependent, similar to the oculomotor recordings. Secondly, the recordings of eye movements will be different depending on the understanding of implicit contents.

## 2. Methods

### 2.1. Participants

We tested 139 children from a primary school in Paris. In total, 34 children belong to the first grade, 25 children belong to the second grade, 29 children belong to the third grade, 28 children belong to the fourth grade, and 23 children belong to the fifth grade. For each child, we evaluated their reading age by using the ELFE test (Évaluation de la Lecture en FluencE) (www.cognisciences.com, Grenoble, France). We presented the text “Monsieur Petit” to each child and he/she was invited to read it aloud for 1 min; the examiner counted the number of words read. Note that the ELFE test is widely employed in French laboratories/clinics for assessing reading age in children and eventual reading deficits [38]. In Table 1, this information is reported. Based on the ELFE test results, five children were excluded, given that their reading age was significantly lower compared to the mean reading age of the other children in the class (one child belonged to the first class, two children to the third grade, and two children to the fourth grade).

### 2.2. Linguistic Materials

Twelve short stories were created, followed by a short sentence containing a specific type of implicit content (presupposition, implicature, and irony). After that, there was a comprehension yes/no question to check whether the child had understood the implicit content in the text (see Appendix A). The type of implicit content presented and the number of correct ‘yes’ and ‘no’ answers were counterbalanced.

### 2.3. Eye Movement Recordings

All participants were tested individually in a soundproof room. They were seated 60 cm from the screen, with a chin rest and a forehead rest. Eye movements were recorded using the Tobii Fusion at a sampling rate of 250 Hz (Tobii Pro, Stockholm, Sweden). The eye tracker was mounted at the bottom of the PC screen on the table facing each participant on which sentences were presented. Prior to starting the reading texts, a nine-point calibration method was used to calibrate both eyes of each child.

### 2.4. Procedure

The child was asked to read aloud short texts that were composed of a context and a target sentence conveying either a presupposition, an implicature, or an ironic meaning. After each text, there was a comprehension yes/no question to check whether the participant has inferred the implicit content from the text. In total, there were twelve contexts, each followed by three target sentences (one containing a presupposition, another containing an implicature, and the last containing irony), i.e., 12 × 3 = 36 texts. The children had to read the thirty-six texts and answer the corresponding comprehension question. Some training texts were presented to the children beforehand to ensure that they understood the task to be carried out. The experiment lasted around 20 min and a brief pause of a few minutes was implemented after reading each of the 12 sentences in order to avoid fatigue.

### 2.5. Data Analysis

Tobii Pro Lab software (Tobii Pro, Stockholm, Sweden) was used to create areas of interest and to measure the number and duration of fixations for each sentence read; in other words, each sentence was considered as an area of interest. We also calculated the number of correct responses obtained by each child. We excluded data from the analysis when fixations fell outside the area of interest, as well as trials including blinks.

### 2.6. Statistical Analysis

A one-way ANOVA was used to compare the reading ages in the different classes. Repeated measure ANOVAs were conducted for the duration and the number of fixations between the correct and wrong responses related to the three different implicit types (presupposition, implicature, and irony) as a within-subjects factor and the different classes of children as a between-subjects factor. Correlations between the ELFE test and the number of corrected responses were examined in the five classes by using the Pearson correlation coefficients. Post-hoc pairwise comparisons were made using the Bonferroni procedure. The effect of a factor was considered significant when the *p*-value was below 0.05. All statistical analyses were processed using JASP software O.17.3 (a free open-source program for statistical analysis supported by the University of Amsterdam, Amsterdam, The Netherlands).

## 3. Results

**Figure 1:**
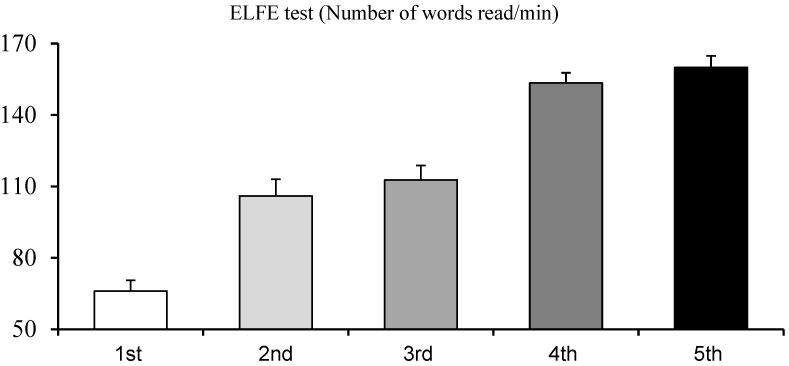
Mean and standard error of the number of words read in 1 min (ELFE test) in the different classes.

The ANOVAs reported a significant class effect for the score of the ELFE test (F_(4,129)_ = 53.15, *p* < 0.001, η^2^ = 0.62). The Bonferroni post hoc test reported a significant difference between the different classes (all *p* < 0.001) except for two comparisons (the second grade vs. the third grade, and the fourth grade vs. the fifth grade), see Figure 1.

**Figure 2:**
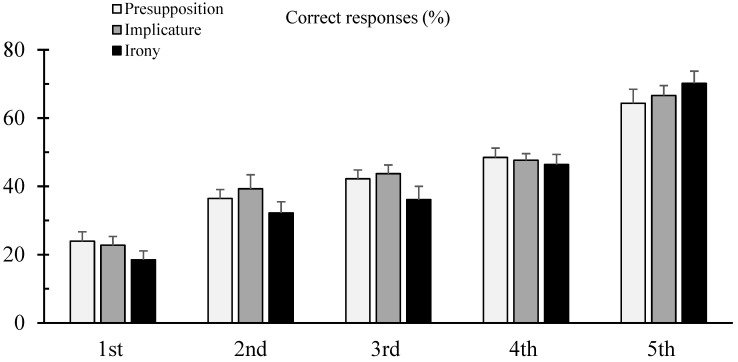
Mean and standard error of the percentage of correct responses for the three different types of implicitness tested (presupposition, implicature, and irony) in the different classes.

Figure 2 shows the percentage of correct responses to the three different types of implicitness (presupposition, implicature, and irony) in the different classes. The ANOVA showed a significant class effect only (F_(4,128)_ = 52.45 *p* < 0.001, η^2^ = 0.48). The Bonferroni results reported significant differences between the classes (all *p* < 0.001) except for two comparisons (the second grade vs. the third grade and the third grade vs. the fourth grade).

A significant positive correlation was also found between the number of words read in 1 min (ELFE test) and the percentage of correct responses in the three different types of implicitness (R = 0.62, *p* < 0.001, R = 0.65, *p* < 0.001, and R = 0.60, *p* < 0.001, respectively, for presupposition, implicature, and irony).

**Figure 3:**
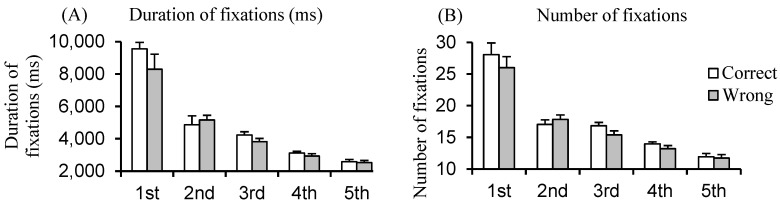
Mean and standard error of the duration (**A**) and the number of fixations (**B**) during reading sentences in the different classes.

Figure 3A,B show the duration and the number of fixations during reading sentences. The ANOVA reported a significant class effect (F_(4,124)_ = 12.59, *p* < 0.001, η^2^ = 0.29 and F_(4,124)_ = 13.93, *p* < 0.001, η^2^ = 0.26, respectively, for the duration and the number of fixations). The post hoc test showed that children in the first class had longer and more frequent fixations than children in the other classes (all *p* < 0.001); the post hoc test also reported that the duration and the number of fixations were significantly longer and more frequent in the second class with respect to the fourth and fifth classes (all *p* < 0.01). The ANOVA also showed a significant effect of the understanding of the implicitness (correct versus wrong) (F_(1,124)_ = 6.52, *p* < 0.01, η^2^ = 0.01 and F_(1,124)_ = 6.98, *p* < 0.01, η^2^ = 0.01, respectively, for the duration and the number of fixations). The duration and number of fixations were significantly longer and more frequent for correct understanding than for incorrect understanding of implicitness. Finally, the ANOVA reported an interaction between the class and understanding (F_(4,124)_ = 4.20, *p* < 0.004, η^2^ = 0.02 and F_(4,124)_ = 3.59, *p* < 0.009, η^2^ = 0.02, respectively, for the duration and number of fixations). Only in children of the first class were the duration and frequency of fixations significantly more important for the sentences correctly understood (*p* < 0.001).

## 4. Discussion

The main findings of this study are as follows: (i) reading time decreases with the age of children and is positively correlated with the accurate understanding of implicit contents; (ii) eye movement patterns when reading texts containing implicitness differ depending on the correct or wrong understanding of these implicit contents; (iii) the three types of implicitness tested in the present study (presupposition, implicature, and irony) are similarly understood by children. In addition, they are better understood by older children (fifth class) than by young children (first class). These results are discussed individually below.

The correlation between reading time and good comprehension of implicitness confirms the results obtained in the 2000s on the relationship between reading fluency and text comprehension in general (that is to say, without the presence of implicit contents). Studies by [39,40,41] have shown that reading time decreases with increasing comprehension. The reasons for this are, on the one hand, that “when strategies for identifying written words are not fast enough, words already read disappear from working memory before subsequent words can be recognized, and this hampers the establishment of links between words and, in so doing, damages text comprehension” (our translation from [42], cf. also by [43]). On the other hand, slow readers read mainly in segments of one or two words. As a result, they are unable to access the syntactic structure of the sentence [44], which is a requisite for gracing the semantic structure of the sentence. A good working memory and access to the semantic sentence structure are essential for understanding the three types of implicitness tested in our study. To understand a presupposition, an implicature, or an ironic sentence, the reader needs to pay attention to the words used (e.g., that “finish” lexically presupposes “having started”; using “carsickness” and “drive for two hours on small bumpy roads” to deduce the correct implicature “he might throw up”) or memorize the context that precedes the ironic sentence to understand that he has to reverse the meaning of this one. The same applies to syntactic and semantic structures, as the presence of a definite determiner, a verb, or an adverb will not trigger the same type of presupposition, as evidenced by the presence of “the” in “Alice wants to help her mother carry her shopping. She takes a packet of tissues and the toothpaste”, which triggers an existential presupposition of the uniqueness of toothpaste, while the presence of the adverbial locution “any more” in “Julie doesn’t like wearing trendy clothes. She prefers wearing large sweaters and old trainers. She never wears skirts or dresses any more” implies the presupposition that Julie once wore dresses. As far as implicatures are concerned, understanding the syntactic grouping of words that together make sense is essential for making a correct inference, because if the reader, for example, makes the following breakdown of the sentence “[He] [has] [to drive] [for] [two] [hours on] [small] [bumpy] [roads to] [go shopping]”, he will not be able to deduce the right implicature, since the sentence will not make sense to him. To understand that a sentence generates an implicature, the reader must be able to extract significant word groups from it, such as “drive for two hours” and “small bumpy roads”. In other words, he must be able to read a sentence without segmenting it into one or two words. Understanding irony is even more demanding in terms of identifying the semantic structure of the ironic sentence, since the reader must be able to determine it on the basis of his or her understanding of the semantic sentence structure that provides the context. Without this understanding, the reader cannot make the inference that the meaning of the ironic sentence must be reversed.

Concerning the difference in eye movement patterns that we observed when the understanding of the implicit contents is satisfactory or unsatisfactory, this study confirms the oculomotor pattern already reported in children (see Section 1). Young children make several fixations of longer duration when they start to read (at about 6 years old); with increasing age, children read more quickly and they decrease the duration and number of fixations. Such changes are related to the development of the cortical structures responsible for eye movements (e.g., the frontal and parietal cortex [45], as well as the temporal and parietal areas involved in linguistic processes [46,47]). Longitudinal fMRI/EEG studies [48] reported that the visual word formation area (located in the left lateral occipitotemporal sulcus) develops with the improvement in reading skills in children from 7 to 12 years old. Based on all these studies, we could assume that such cortical structures, controlling both the triggering of eye movements and reading performance, follow a similar development. While this oculomotor pattern has been reported in children reading different types of languages (English, [31]; French, [33]; Italian, [49]; and German, [50]), suggesting that the eye movement pattern in children follows a developmental trajectory irrespective of the orthography of the language, no studies, to the best of our knowledge, exist in which the oculomotor pattern is investigated in relationship to the understanding of implicitness. This is the novelty of the present study. The finding that the duration and number of fixations are significantly higher when children understood the implicitness correctly is in line with the hypothesis that during fixation, the cortical process and working memory are acting together in order to understand the words read by the child; consequently, if the child does not take enough time to perform these processes, he does not correctly understand the word he is reading. Interestingly, we showed that this occurs particularly in young children (in the first grade); in other words, a child starting to read and understand implicit content needs more time to fixate on the word in order to understand its meaning (see Section 1).

Children’s ability to understand presuppositions, as well as implicatures or ironic sentences, corroborates the results already obtained by studies already carried out on this subject. Although each study has focused on only one of the types of implicitness analyzed here, they all converge in showing that all three kinds of implicitness can be understood by children as young as 5 years old. Some studies even show that from the age of 3–4, a child would be able to understand some scalar implicatures [16] or some ironic sentences [26,27]). The finding that older children understand these three types of implicit content better than younger children also confirms the results obtained in previous studies, which all show that from the age of eight (second grade), children are more likely to understand the implicit content than younger children and that this mastery in understanding improves with age.

## 5. Conclusions

Given the importance of implicitness in obtaining a coherent representation of a text, the results obtained in this study argue in favor of the possibility of teaching the comprehension of some types of implicitness (presupposition, implicature, and irony) from an early age. A previous study [2] showed that readers (children or adults) were able to give correct answers when faced with the same corpus of texts studied in this paper, however without detecting the triggers of presupposition, implicature, or irony that generate these implicit contents. It follows that the duration and number of fixations on the words (presupposition), groups of words (implicature), or context (irony) that act as triggers seem to play no relevant role in determining whether the pupils’ response to the given question will be correct or not. This issue deserves to be examined in a future study.

## 6. Practical Implications

The results obtained in this study, which shows that reading speed is correlated with adequate comprehension of implicit meaning, suggest that time for reading should be set aside in class to allow each pupil to read a book of their choice individually. If practiced every day, this kind of exercise would enable pupils to increase their reading speed over the days and, as a result, acquire a better mastery of the implicit contents.

However, this type of teaching should also be combined with reading workshops in small groups (or with the whole class), during which the teacher reads a children’s book and then questions pupils about the book’s parts that generate implicitness. In doing so, the teacher teaches pupils to produce inferences that enable them to better understand word meanings (the verb ‘to manage to’ presupposes ‘to try’); to understand the logic that governs the sequence of sentences (given that Tom is a bad pupil, his parents can be pleased even if he has only passed two exercises; a case of implicature); and the coherence that paradoxically emerges from the juxtaposition of two antithetical sentences (Lily prefers wearing large sweaters and old trainers. Her brother says to her: “Wow, you dress like a princess!”; a case of irony). By teaching pupils to make the hidden meanings of some words explicit in this way, the teacher would be completing the pupils’ vocabulary learning in another way, since it would no longer be a matter of only explaining words that are considered complicated, but of making pupils aware of the implications of everyday words. As a result, the teacher would make pupils responsible for their own words, to which no one could object: “Of course you did not manage, because you did not even try!”. Regarding implicatures, which enable us to understand the internal structure of the narrative, since their function in literature is to manage the suspense of the story in a hidden way [51], learning to master them would enable pupils to increase their enjoyment of reading by being able to make hypotheses (which may or may not be validated) about how the story will unfold. In this way, the teacher can turn pupils into active readers who are capable of imagining the events that might occur depending on the part of the book they are reading. Understanding irony plays a role in pupils’ social integration over the long term, as shown by [52], which highlights that irony is used in conversations between young adults or friends every two minutes on average. This type of training (individual silent reading and explicit comprehension workshops with the teacher) also needs to be thought through over the long term, as learning to understand implicit meaning takes time and also depends on the brain’s maturity. It is therefore not possible to teach implicitness understanding over a short period of time, as can be done when teaching children to add. What is more, we need to adapt to the cognitive abilities of pupils, and therefore draw on the work of neurolinguists and psycholinguists to determine what type of implicitness can be taught at what age. Learning to understand presupposition, implicatures, and irony can begin as early as 5 years old, whereas argumentative implicitness, linked to the use of connectors such as ‘but’, needs to wait until the pupil is at least 11–12 years old.

## Figures and Tables

**Table 1:** Mean and standard deviation of age (years) and number of words read in 1 min (the ELFE test) for children belonging to the different primary classes.

	Number of Children Tested	Age (Years)	ELFE Test (N Words Read/min)
First grade	33	6.8 ± 0.2	66.0 ± 4.5
Second grade	25	7.9 ± 0.1	106 ± 7.0
Third grade	27	9.0 ± 0.1	112 ± 6.1
Fourth grade	26	10.0 ± 0.1	153 ± 4.2
Fifth grade	23	11.0 ± 0.1	160 ± 4.7

## Data Availability

The original contributions presented in this study are included in the article. Further inquiries can be directed to the corresponding author.

## Appendix A

**Conditions**	**Type**	**Common** **Context**	**Target** **Sentence** **and Triggers**	**Question with the Correct** **Answer**
Presupposition	Definite description	Amber wants to help her mother carry her shopping.	She takes a packet of tissues and the toothpaste.	In your opinion, were there several toothpastes? No
Implicature	Quantitative	Amber wants to help her mother carry her shopping.	She takes a packet of tissues and the toothpaste.	In your opinion, did Amber only take the packet of tissues and the toothpaste? Yes
Irony	Antithetical	Amber wants to help her mother carry her shopping.	She takes a packet of tissues. Her mother says to her: “How strong you are”	In your opinion, does the mother think that Alice is strong? No
Presupposition	Aspectual adverb	Florence usually arrives late for her appointments. When invited to have dinner with friends, she arrives two hours late and explains that she has had a problem.	She wanted to catch the 8h05 train, but it was canceled again.	In your opinion, is it the first time that the 8.05 p.m. train is cancelled? No
Implicature	Quantitative	Florence usually arrives late for her appointements. When invited to have dinner with friends, she arrives two hours late and explains that she had a problem.	She’s not usually that late.	In your opinion, has she ever arrived this late before? Yes
Irony	Antithetical	Florence usually arrives late for her appointments. When invited to have dinner with friends, she arrives two hours late and explains that she has had a problem.	Someone says: “How surprising!”	In your opinion, is this person very surprised? No
Presupposition	Aspectual verb	Tom does not like school. He has a lot of bad grades. This weekend, his parents have to sign his exercise book.	They are pleased because Tom continues to make progress in arithmetics.	In your opinion, is this the first time that Tom makes progress in arithmetics? No
Implicature	Scalar	Tom does not like school. He has a lot of bad grades. This weekend, his parents have to sign his exercise book.	They are pleased because Tom got two exercises right.	In your opinion, has he failed all the other exercises? Yes
Irony	Antithetical	Tom does not like school. He has a lot of bad grades. This weekend, his parents have to sign his exercise book.	His father says “so, still top of the class, are you?”	In your opinion, does Tom’s father think his son is top of the class? No
Presupposition	Aspectual adverb	Lily does not like wearing trendy clothes. She prefers wearing large sweaters and old trainers.	She never wears skirts or dresses any more.	In your opinion, has Lily ever worn skirts or dresses? Yes
Implicature	Scalar	Lily does not like wearing trendy clothes. She prefers wearing large sweaters and old trainers.	She wears most of her cousin’s cast-offs.	In your opinion, are there any of her cousin’s clothes that Lily isn’t getting back? Yes
Irony	Antithetical	Lily does not like wearing trendy clothes. She prefers wearing large sweaters and old trainers.	Her brother says to her: “Wow, you dress like a princess!”	In your opinion, does her brother think that Lily dresses like a princess? No
Presupposition	Factive verb	Oliver suffers from carsickness. He has to drive for two hours on small bumpy roads to go shopping.	He regrets living so far from the town.	In your opinion, does he live close to a town? No
Implicature	Scalar	Oliver suffers from carsickness. He has to drive for two hours on small bumpy roads to go shopping.	He might throw up.	In your opinion, it is certain that he will throw up? No
Irony	Antithetical	Oliver suffers from carsickness. He has to drive for two hours on small bumpy roads to go shopping.	He says “What a lucky guy I am!”	In your opinion, is Oliver cross about taking the car? Yes
Presupposition	Implicative verb	Lea comes home after a tennis tournament. She is very hungry; she looks at what is on the table and says:	Thank you Mummy, but I don’t think I’ll manage to eat the six sausages.	In your opinion, is Lea going to try to eat the six sausages? Yes
Implicature	Scalar	Lea comes home after a tennis tournament. She is very hungry; she looks at what is on the table and says:	Thank you Mummy, but six sausages are a bit much!	Do you think there are more than six sausages on the table? No
Irony	Antithetical	Lea comes home after a tennis tournament. She is very hungry; she looks at what is on the table and says:	Great, Mummy has made my favorite dish: burnt nuggets!	In your opinion, does Léa love burnt nuggets? No
Presupposition	Aspectual adverb	Max and Harry go to a restaurant. On the menu, there is chocolate mousse and strawberry tarts, but Harry wants a strawberry mousse.	He loves mousses but has discovered that he is allergic to chocolate and he mustn’t eat chocolate any more.	In your opinion, has Harry ever eaten chocolate before? Yes
Implicature	Scalar	Max and Leo go to a restaurant. On the menu, there is chocolate mousse and strawberry tarts, but Leo wants a strawberry mousse.	Max tries to explain to him that this will not be possible.	Do you think Leo will understand? No
Irony	Antithetical	Max and Leo go to a restaurant. On the menu, there is chocolate mousse and strawberry tarts, but Leo wants a strawberry mousse.	Max says to him: “You’re such an uncomplicated guy.”	In your opinion, does Max think Leo is complicated? Yes
Presupposition	Factive connector	Sonny wants to play cards. He asks Victoria to play with him. She says that she prefers playing all by herself.	But since it is Sonny’s birthday, she agrees for once.	In your opinion, is it Sonny’s birthday today? Yes
Implicature	Scalar	Sonny wants to play cards. He asks Victoria to play with him. She says that she prefers playing all by herself.	But since it is Sonny’s birthday, she agrees for once.	In your opinion, does Victoria generally refuse to play with Sonny? Yes
Irony	Antithetical	Sonny wants to play cards. He asks Victoria to play with him. She says that she prefers playing all by herself.	Sonny says; “That’s nice of you”	In your opinion, does Sonny think that Victoria is not nice with him? Yes
Presupposition	Aspectual adverb	Daniel and his family are going to the seaside. While getting off the train, Daniel falls and breaks his arm.	He will have to postpone his sailing camp again.	In your opinion, had Daniel already postponed his sailing camp in the past? Yes
Implicature	Scalar	Daniel and his family are going to the seaside. While getting off the train, Daniel falls and breaks his arm.	It will be painful for a while but not for very long.	In your opinion, will this be very painful for Lucas? No
Irony	Antithetical	Daniel and his family are going to the seaside. While getting off the train, Daniel falls and breaks his arm.	His father says: “You’re going to have a fun week!”	In your opinion, does his father think that Daniel is going to be bored this week? Yes
Presupposition	Factive verb	Diana and Luke are on a hike. At the campsite in the evening, Diana realises that she has lost the bag with the food and that there is only a packet of dried soup left.	And also, she does not know that Luke hates soup.	In your opinion, does Luke like soup? No
Implicature	Clausal	Diana and Luke are on a hike. At the campsite in the evening, Diana realises that she has lost the bag with the food and that there is only a packet of dried soup left.	If Luke hates soup, he will be very disappointed.	In your opinion, are we sure that Luke hates soup? No
Irony	Antithetical	Diana and Luke are on a hike. At the campsite in the evening, Diana realises that she has lost the bag with the food and that there is only a packet of dried soup left.	Luke says: I see we’re in for a feast this evening.	In your opinion, does Luke think that they are in for a feast? No
Presupposition	Aspectual verb	Alexander has bought a big house in the country. There is a lot of work to do on it.	He has still not finished painting the walls.	In your opinion, has he started painting the house? Yes
Implicature	Aspectual verb	Alexander has bought a big house in the country. There is a lot of work to do on it.	A week ago, he started painting the walls	In your opinion, has he finished painting the walls? No
Irony	Antithetical	Alexander has bought a big house in the country. There is a lot of work to do on it.	Seeing how clumsy he is, it will be quick work.	In your opinion, will the works on the house take a long time? Yes
Presupposition	Factive verb	Martin hates his aunt who is always very mean and very harsh towards him. This year, Martin’s parents tell him that his aunt won’t be able to come for Christmas.	He is very pleased because he thinks that his aunt will give him a Christmas present anyway.	In your opinion, will Martin receive a present from his aunt this year? No
Implicature	Quantitative	Martin hates his aunt who is always very mean and very harsh towards him. This year, Martin’s parents tell him that his aunt won’t be able to come for Christmas.	She has broken her leg.	In your opinion, did Martin’s aunt only break her leg? Yes
Irony	Antithetical	Martin hates his aunt who is always very mean and very harsh towards him. This year, Martin’s parents tell him that his aunt will not be able to come for Christmas.	Martin says: “oh, what a pity!”	In your opinion, is Martin pleased? Yes

## References

[1] Van Djik T.A., Kintsch W. (1983). Strategies of Discourses Comprehension.

[2] Pozniak C., Beyssade C., Roussarie L., Godart-Wendling B. (2024). How Relevant Is the Sentence Unit to Accessing Implicit Meaning?. Languages.

[3] Grice P. (1975). Logic and Conversation. Syntax and Semantics 3: Speech Acts, Peter Cole and Jerry Morgan.

[4] Garmendia J. (2018). Irony.

[5] Berger F., Höhle B. (2012). Restrictions on addition: Children’s interpretation of the focus particles auch ‘also’ and nur ‘only’ in German. J. Child Lang..

[6] Matsuoka K., Miyoshi N., Hoshi K., Ueda M., Yabu I., Hirata M., Bamman D., Magnitskaia T., Zaller C. (2006). The acquisition of Japanese focus particles: Dake ‘only’ and mo ‘also’. Proceedings of the 30th Boston University Conference on Language Development Supplement.

[7] Kail M. (1978). La comprehension des présuppositions chez l’enfant. L’année Psychol..

[8] Bergsma W. (2006). (Un)Stressed ook in Child Dutch. Semantics in Acquisition.

[9] Höhle B., Berger F., Müller A., Schmitz M., Weissenborn J. (2009). Focus particles in children’s language: Production and comprehension of auch ‘also’ in German learners from 1 year to 4 years of age. Lang. Acquis..

[10] Hüttner T., Drenhaus H., van de Vijver R., Weissenborn J. The acquisition of the German focus particle auch ‘too’: Comprehension does not always precede production. Proceedings of the 28th Annual Boston University Conference on Language Development.

[11] Nederstigt U. (2003). Auch and Noch in Child and Adult German.

[12] Domaneschi F., Di Paola S., Pouscoulous N. (2022). The development of presupposition: Pre-schoolers’ understanding of *regret* and *too*. Intercult. Pragmat..

[13] Scoville R., Gordon A. (1980). Children’s understanding of factive presuppositions: An experiment and a review. J. Child Lang..

[14] Papafragou A., Tantalou N. (2004). Children’s Computation of Implicatures. Lang. Acquis..

[15] Pouscoulous N., Noveck I., Politzer G., Bastide A. (2007). A Developmental Investigation of Processing Costs in Implicature Production. Lang. Acquis..

[16] Stiller A., Goodman N., Franck M. (2015). Ad-hoc Implicature in Preschool Children. Lang. Learn. Dev..

[17] Eiteljoerge S., Pouscoulous N., Lieven E. (2018). Some Pieces Are Missing: Implicature Production in Children. Front. Psychol..

[18] Foppolo F., Chierchia G. (2012). Scalar Implicatures in Child Language: Give Children a Chance. Lang. Learn. Dev..

[19] Noveck I.A. (2001). When children are more logical than adults: Experimental investigations of scalar implicature. Cognition.

[20] Singh R., Wexler K., Astle-Rahim A., Kamawar D., Fox D. (2016). Children interpret disjunction as conjunction: Consequences for theories of implicature and child development. Nat. Lang. Seman..

[21] Papafragou A., Musolino J. (2003). Scalar implicatures: Experiments at the semantics-pragmatics interface. Cognition.

[22] Tieu L., Yatsushiro K., Cremers A., Romoli J., Sauerland U., Chemla E. (2016). On the Role of Alternatives in the Acquisition of Simple and Complex Disjunctions in French and Japanese. J. Semant..

[23] Aguert M., Le Vallois C., Martel K., Laval V. (2018). That’s really clever!” Ironic hyperbole understanding in children. J. Child Lang..

[24] Rivière F., Klein M., Champagne-Lavau M. (2018). Using context and prosody in irony understanding: Variability amongst individuals. J. Pragmat..

[25] Massaro D., Valle A., Marchetti A. (2014). Do social norms, false belief understanding, and metacognitive vocabulary influence irony comprehension? A study of five- and seven-year-old children. Eur. J. Dev. Psychol..

[26] Loukusa S., Leinonen E. (2008). Development of comprehension of ironic utterances in 3- to 9-year-old finnish-speaking children. Psychol. Lang. Commun..

[27] Angeleri R., Airenti G. (2014). The Develoment of Joke and Irony Understanding: A Study with 3- to 6-year-old Children. Can. J. Exp. Psychol..

[28] Rayner K. (1998). Eye Movements in Reading and Information Processing: 20 Years of Research. Eye Mov. Read..

[29] Levy-Schoen A., O’Regan J., Kolers H.B.P.A., Wrolstand M.E. (1979). The control of eye movements in reading. Processing of Visible Language.

[30] Buswell G. (1922). Fundamental Reading Habits: A Study of Their Development.

[31] McConkie G.W., Zola D., Grimes J., Kerr P.W., Bryant N.R., Wolff P.M., Stein J.F. (1991). Children’s eye movements during reading. Vision and Visual Dyslexia.

[32] Rayner K., Slattery T.J., Drieghe D., Liversedge S.P. (2011). Eye movements and word skipping during reading: Effects of word length and predictability. J. Exp. Psychol. Hum. Percept. Perform..

[33] Seassau M., Bucci M.P. (2013). Reading and visual search: A developmental study in normal children. PLoS ONE.

[34] Kanga S.N., Yimb D. (2018). Reading Comprehension and Reading Processing of School-Aged Children with Specific Language Impairment Using Eye Tracker. Commun. Sci. Disord..

[35] Farsani D., Radmehr F., Alizadeh M., Zakariya Y.F. (2021). Unpacking the black-box of students’ visual attention in mathematics and English classrooms: Empirical evidence using mini-video recording gadgets. J. Comput. Assist. Learn..

[36] Olkoniemi H., Halonen S., Pexman P.M., Tuomo Haikio T. (2023). Children’s processing of written irony: An eye-tracking study. Cognition.

[37] Au-Yeung S.K., Kaakinen J.K., Liversedge S.P., Benson V. (2015). Processing of Written Irony in Autism Spectrum Disorder: An Eye-Movement Study. Autism Res..

[38] Caldani S., Gerard C.L., Peyre H., Bucci M.P. (2020). Visual Attentional Training Improves Reading Capabilities in Children with Dyslexia: An Eye Tracker Study During a Reading Task. Brain Sci..

[39] Jenkins J.R., Fuchs L.S., van den Broek P., Espin C., Deno L. (2003). Sources of individual differences in reading comprehension and reading fluency. J. Educ. Psychol..

[40] Rasinski T., Flexer C., Szypulski T. (2006). The Sound of Learning. Why Self-Amplication Matters.

[41] Rasinski T.V. (2010). The Fluent Reader: Oral and Silent Reading Strategies for Building Word Recognition, Fluency, and Comprehension.

[42] Dubé F., Bessette L., Ouellet C. (2016). Développer la fluidité et la compréhension en lecture afin de prévenir les difficultés. La Nouv. Rev. de L’adaptation et de la Scolarisation.

[43] Kirby R.J. (2007). Reading comprehension: Its nature and development. Encyclopedia of Language and Literacy Development.

[44] Daane M.C., Campbell J.R., Grigg W.S., Goodman M.J., Oranje A. (2005). Fourth-Grade Students Reading Aloud: NAEP 2002 Special Study of Oral Reading.

[45] Luna B., Velanova K., Geier C.F. (2008). Development of eye-movement control. Brain Cogn..

[46] Simos P.G., Breier J.I., Fletcher J.M., Foorman B.R., Mouzaki A., Papanicolaou A.C. (2001). Age-related changes in regional brain activation during phonological decoding and printed word recognition. Dev. Neuropsychol..

[47] Turkeltaub P.E., Gareau L., Flowers D.L., Zeffiro T.A., Eden G.F. (2003). Development of neural mechanisms for reading. Nat. Neurosci..

[48] Hannagan T., Amedi A., Cohen L., Dehaene-Lambertz G., Dehaene S. (2015). Origins of the specialization for letters and numbers in ventral occipitotemporal cortex. Trends Cogn. Sci..

[49] De Luca M., Di Pace E., Judica A., Spinelli D., Zoccoloti P. (1999). Eye movement patterns in linguistic and non-linguistic tasks in developmental surface dyslexia. Neuropsychologia.

[50] Trauzettel-Klosinski S., Koitzsch M., Durrwachter U., Sokolov A.N., Reinhard J., Klosinski G. (2010). Eye movements in German-speaking children with and without dyslexia when reading aloud. Acta Ophthalmol..

[51] Baroni R. (2017). Les Rouages de L’intrigue.

[52] Gibbs R.W. (2000). Irony in Talk Among Friends. Metaphor. Symb..

